# Real-World Data on Potent P2Y12 Inhibition in Patients with Suspected Chronic Coronary Syndrome, Referred for Coronary Angiography

**DOI:** 10.1159/000527459

**Published:** 2022-10-10

**Authors:** Erik Träff, Dimitrios Venetsanos, Karin Alpkvist, Sofia Sederholm Lawesson, Mikolaj Skibniewski, Sammy Zwackman, Joakim Alfredsson

**Affiliations:** ^a^Department of Health, Medicine and Caring Sciences and Department of Cardiology, Linköping University, Linköping, Sweden; ^b^Division of Cardiology, Department of Medicine, Karolinska Institute Solna and Karolinska University hospital, Stockholm, Sweden

**Keywords:** Stable angina, Chronic coronary syndrome, Platelet, Bleeding, Percutaneous coronary intervention

## Abstract

**Introduction:**

Potential benefit with potent platelet inhibition in patients with chronic coronary syndrome (CCS) undergoing percutaneous coronary intervention (PCI) has been discussed. The aim of this study was to compare a potent P2Y12 inhibition strategy using ticagrelor with clopidogrel in CCS patients referred for coronary angiography (CA) and PCI if feasible.

**Methods:**

In this retrospective real-world study, patients referred for outpatient CA due to suspected CCS were included. To adjust for group differences, a propensity score reflecting the probability of being treated with ticagrelor was calculated and added to the logistic regression outcome model.

**Results:**

In total, 1,003 patients were included in the primary analysis (577 treated with clopidogrel and 426 with ticagrelor). Among clopidogrel-treated patients, 132 (22.9%) experienced a bleeding complication compared with 93 (21.8%) among ticagrelor-treated patients, with no significant difference between the groups (*p* = 0.70). There was no difference in bleeding severity. Furthermore, we observed no statistically significant difference in major adverse cardiovascular events (MACE [death, stent thrombosis, myocardial infarction, or stroke]) (1.2% vs. 2.3%, *p* = 0.17). A subgroup analysis restricted to patients undergoing PCI ad hoc displayed a similar pattern. Also, patients undergoing CA without PCI ad hoc frequently experienced a bleeding complication, with no difference between the two treatments (21.0% vs. 17.3%, *p* = 0.27). Propensity score adjusted analyses confirmed the results.

**Discussion:**

In patients with CCS referred for CA and PCI if feasible, a more potent P2Y12 inhibition strategy with ticagrelor was not associated with bleeding complications or MACE compared with clopidogrel.

## Introduction

From the Euro Heart Survey on coronary revascularization, chronic coronary syndrome (CCS) was reported as the most frequent indication for coronary angiography (CA) and subsequent percutaneous coronary intervention (PCI), which was often performed ad hoc [1]. The latest European Society of Cardiology guidelines on revascularization state that ad hoc PCI is convenient, often cost-effective, safe, and associated with fewer access site complications and lower radiation exposure [2].

The introduction of dual antiplatelet therapy (DAPT) with aspirin and P2Y12-inhibitors has reduced the risk of thrombotic occlusions of the coronary arteries in patients undergoing PCI [3, 4]. Registry studies have suggested significantly lower occurrence of thrombotic events with a more efficient platelet inhibition at the time of PCI, based on early preloading with clopidogrel compared with immediately after stenting [5]. In line with this, a higher loading dose of clopidogrel reduced periprocedural myocardial infarction (MI) [6]. In contrast, two randomized trials comparing a clopidogrel loading dose administered prior to diagnostic CA versus administration in the catheterization laboratory immediately prior to PCI, found no significant difference in ischemic outcomes [7, 8]. However, there is a large interindividual variation in the effect of clopidogrel, based on metabolic genetic differences, and the onset of the effect of clopidogrel is much slower than later, more potent P2Y12-inhibitors, indicating that a significant proportion of these clopidogrel-treated patients, even if administered before angiography, had a low degree of platelet inhibition at the time of PCI [9].

Over time, rates of PCI-associated stent thrombosis (ST), stroke, and MI have decreased substantially. Nevertheless, the use of high-sensitive troponin (hs-Tn) assays has shown a persistent and frequent occurrence of periprocedural hs-Tn elevation. These, often asymptomatic, myocardial injuries have been associated with worse outcomes, including major adverse cardiac events (MACE) and death [10, 11, 12].

In acute coronary syndrome patients undergoing PCI, more potent platelet inhibition with ticagrelor or prasugrel have improved outcome compared with clopidogrel and are now strongly recommended in clinical guidelines [13, 14, 15, 16]. Therefore, a potential benefit from a more potent platelet inhibition at the time of PCI could be anticipated also in patients with CCS treated with PCI. Intravenous P2Y12 inhibition at the time of PCI has shown some benefits but with more bleeding complications [17]. In contrast, two recently published randomized trials on mainly elective patients undergoing PCI indicated no benefit with potent platelet inhibition, with ticagrelor or prasugrel, respectively, as compared with clopidogrel, administered immediately before PCI [18, 19]. However, also more potent-platelet inhibitors, orally and conventionally administered (as a whole tablet) immediately before PCI, will lack significant effects on platelet activity at the time of PCI [9]. There is a lack of data regarding the use of more potent platelet inhibition in patients with suspected CCS referred for CA, administered in a timely fashion to achieve sufficient platelet inhibition at the time of the procedure.

The primary aim of this study was to evaluate the safety (with respect to bleeding complications) of a strategy based on pretreatment with the more potent P2Y12 inhibitor ticagrelor compared with clopidogrel, in a cohort of real-world patients referred for outpatient CA due to suspected CCS and PCI ad hoc if feasible. The second aim was to assess efficacy. Both drugs were timely administered to reach the anticipated effect at the time of PCI.

## Material and Methods

### Study Population

We used the Swedish Web-System for Enhancement and Development of Evidence-Based Care in Heart Disease Evaluated According to Recommended Therapies (SWEDEHEART) registry to identify patients referred for outpatient CA due to suspected CCS at Linköping University Hospital between April 2010 and February 2015. Linkoping University Hospital is a tertiary referral center with two referring hospitals. All patients were referred for CA after seeing a senior cardiologist at the outpatient clinic. The indication for CA was suspected CCS/angina pectoris in all referred patients. During the study period, the vast majority of CAs due to suspected CCS were on an outpatient basis.

In October 2012, a clopidogrel-based treatment strategy (with loading dose the evening before scheduled CA) was changed to a ticagrelor-based treatment strategy (with a loading dose on arrival to the clinic, the same morning as CA was performed) in addition to aspirin. Based on previous studies, it could be assumed that both drugs had reached a plateau of platelet inhibition after the loading dose [9]. Another reason for the change in clinical routine was that it was believed to be more convenient for the patient to receive the loading dose on arrival to the outpatient clinic.

In this analysis, we included patients with suspected CCS referred for outpatient CA and treated with aspirin and a P2Y12 inhibitor before CA. Patients were excluded if they were not residents of the PCI center's catchment area (and therefore not possible to track in the electronic medical records) and if coronary artery bypass grafting surgery (CABG) was planned during the 30-day follow-up period.

The SWEDEHEART is a nationwide registry including nearly all patients admitted to hospital because of symptoms suggestive of an acute coronary syndrome and all patients undergoing coronary catheterization or heart surgery in Sweden (http://www.ucr.uu.se/swedeheart/). The registry enables online registration by caregivers of around 110 variables for all patients admitted to the hospital and around 250 variables for patients undergoing PCI. Information about patient demographics, medical history, medication before admission, interventions and pharmacological treatment during hospital stay, inhospital outcomes and complications, discharge diagnosis, and discharge medication are prospectively collected. In addition to SWEDEHEART data, using a predefined template, all patients' electronic medical records were searched in detail for inclusion and exclusion criteria, information on baseline characteristics, bleeding history (actionable sign of hemorrhage, requiring consulting with a health care professional, i.e., at least BARC type 2), medication before and after the CA, and complications following the CA. Data from the medical records were merged with data from the SWEDEHEART database. This analysis was performed and presented in accordance with the STROBE statement (http://www.strobe-statement.org/).

### Outcomes

Our primary outcome measure was bleeding events within 30 days after CA. We assessed bleeding complications using two well-established bleeding definitions, the Thrombolysis in Myocardial Infarction (TIMI) and Bleeding Academic Research Consortium (BARC) definitions [20, 21]. A detailed description of bleeding definitions is presented in the supplement. We also assessed 30 days of MACE, defined as the composite of death, ST, MI, and stroke, and the individual components of MACE. ST was defined as angiographically confirmed ST. Periprocedural MI (triggered by symptoms or signs indicative of ischemia) and MI during 30 days of follow-up were defined according to the universal definition of MI [22]. Periprocedural stroke was defined as a neurological deficit caused by an ischemic or hemorrhagic event within 48 h from angiography, with a duration of the neurological deficit of more than 24 h.

### Statistics

Continuous variables are presented as means and standard deviations. Categorical variables are presented as counts and percentages. Comparisons between groups were performed using the χ^2^ test or Fisher's exact test as appropriate.

First, in the main analysis, we performed an as-treated analysis comparing patients treated with ticagrelor and clopidogrel. Two subgroup analyses were performed, restricted to patients undergoing ad hoc PCI and patients undergoing CA alone or CA with a diagnostic procedure. Logistic regression analyses were performed to assess the treatment associations with bleeding, MACE (and the individual components of MACE). A propensity score (PS) was calculated, reflecting the probability of a patient being treated with ticagrelor, and was included in the logistic regression models to adjust for differences in baseline characteristics. The PS model included age, sex, history of MI, PCI, CABG, diabetes mellitus, hypertension, hyperlipidemia, previous bleeding, and treatment with OAC. In addition, creatinine and hemoglobin levels, catheterization access site, and use of drug eluting stent or rotablator were included. Data are presented as odds ratios with 95% confidence intervals.

Second, we performed a sensitivity analysis as an “intention to treat” analysis based on the two time periods when clopidogrel and ticagrelor, respectively, were recommended as first-line treatment. Again, two subgroup analyses were performed, restricted to patients undergoing PCI ad hoc and patients undergoing CA alone.

A *p* value <0.05 was considered statistically significant. All statistical analyses were performed with the SPSS version 23.0 (PASW Statistics 23) software (SPSS, Inc, Chicago, IL).

## Results

In the primary analysis, 1,003 patients met the inclusion and exclusion criteria (577 treated with clopidogrel and 426 treated with ticagrelor). There was no significant difference in age, sex, or medical history. Patients treated with clopidogrel had a higher incidence of chronic kidney disease and anemia. Almost two-thirds of the patients were in Canadian Cardiovascular Society angina class 2, and about one-third were in CCS class 3 (*p* = ns**)** (Table 1).

At angiography, radial access was more common in the ticagrelor group (70% vs. 78% *p* = 0.02). There was no significant difference in severity of coronary disease, with about 30% having 1-vessel disease and about 25% having 3-vessel or left main disease. About 25% of patients had no significant coronary artery disease. We observed no significant difference in PCI performed ad hoc (42.3% vs. 46.9% in clopidogrel and ticagrelor treated patients, respectively), but drug eluting stents (DES) were more often used in patients treated with ticagrelor. The number of patients treated with a rotablator procedure was low, but it was used significantly more often in the ticagrelor group (Table 2). At discharge, there was no significant difference in medication except that more patients in the clopidogrel group were discharged on clopidogrel and more patients in the ticagrelor group were discharged on ticagrelor (Table 2).

### Outcomes

**Fig. 1:**
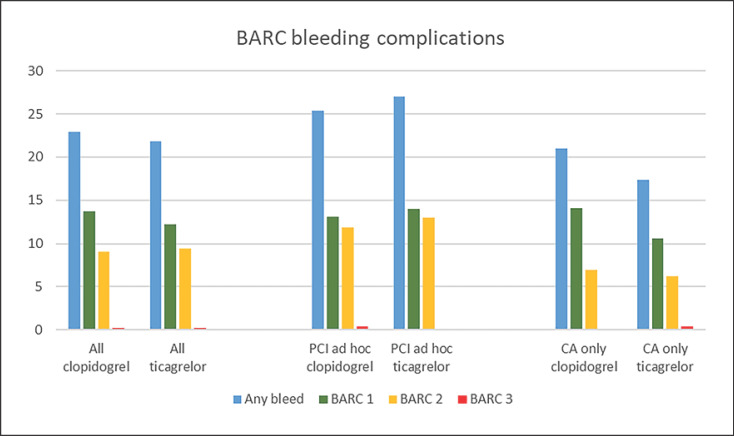
BARC bleeding complications (in percent) in the whole study population, the subgroup undergoing ad hoc percutaneous coronary intervention (PCI) and the subgroup undergoing coronary angiography (CA) only. There were no significant differences between clopidogrel and ticagrelor.

More than 20% of the patients experienced a bleeding complication but without any significant difference between the groups (22.9 vs. 21.8% in clopidogrel and ticagrelor treated patients, respectively). We did not find any difference in the severity of bleeding complications (the majority were TIMI minimal or BARC type 1). The observed bleeding complications were primarily related to the access site, 97.0 versus 97.8% in the clopidogrel and ticagrelor groups, respectively (Table 3). There was no statistically significant difference in MACE (1.2% vs. 2.3%, *p* = 0.17), in clopidogrel and ticagrelor-treated patients, respectively, which almost exclusively consisted of periprocedural MI (shown in Fig. 1 and Table 3).

### Subgroup of Patients Treated with Ad Hoc PCI

A subgroup analysis restricted to patients treated with ad hoc PCI displayed a similar pattern, confirming the results from the whole study population. Even though we found a somewhat higher rate of bleeding complications (25.4 vs. 27.0%) and more severe bleeds, there was no significant difference between clopidogrel and ticagrelor-treated patients. Again, we found no difference in MACE between the two groups (shown in Fig. 1 and Table 4).

### Subgroup of Patients Undergoing CA Only

A substantial number of patients undergoing CA only or CA with a diagnostic procedure experienced a bleeding complication (21.0 vs. 17.3%, *p* = 0.27), with a significant proportion of TIMI minor or BARC type 2, but again with no statistically significant difference between the two treatments. Only one of the 17 MACE occurred among patients undergoing CA only (shown in Fig. 1 and Table 4).

### Adjusted Analyses

**Fig. 2:**
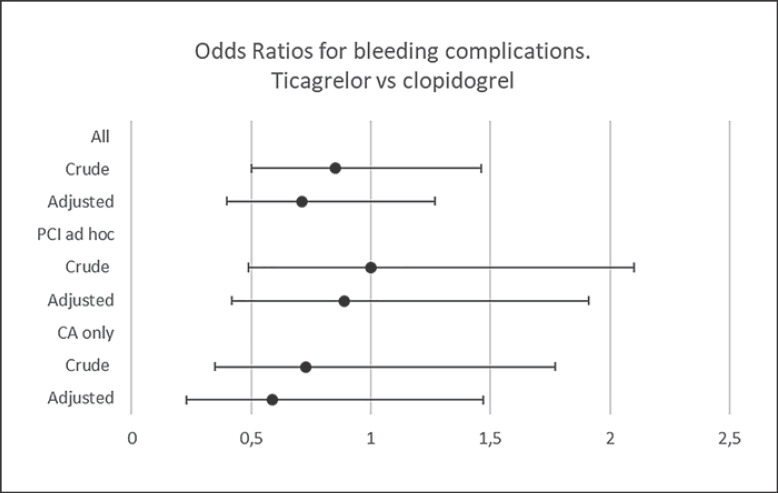
Odds ratio (OR) for any bleeding complication in patients treated with ticagrelor versus clopidogrel (<1 favors ticagrelor). Crude and propensity score-adjusted OR and 95% confidence intervals are presented for the whole population, the subgroup undergoing percutaneous coronary intervention (PCI) ad hoc and the subgroup undergoing coronary angiography (CA) only.

To adjust for baseline differences between the two groups, a propensity score-adjusted analysis was performed, confirming the unadjusted results (shown in Fig. 2).

### Sensitivity Analyses

In a sensitivity analysis, patients were allocated according to an “intention-to-treat” approach based on the recommended first-line P2Y12 inhibitor at the time, i.e., all 502 patients during the clopidogrel-based period and 502 patients during the ticagrelor-based period, as described in the methods section. By study design, in the clopidogrel-based arm, 97.4% were treated with clopidogrel and 2.4% with ticagrelor; in contrast, in the ticagrelor-based period, 82.5% were treated with ticagrelor and 17.5% with clopidogrel. The two study groups were well-balanced in baseline characteristics (including hemoglobin and creatinine) (online suppl. Table S1; see www.karger.com/doi/10.1159/000527459 for all online suppl. material). Notably, more patients underwent PCI ad hoc during the ticagrelor period, and a higher proportion were treated with stents/DES (online suppl. Table S2). The outcome results of the sensitivity analyses confirmed the results of the main analysis, with no significant difference between the two treatments (online suppl. Table S3, S4).

## Discussion

The major finding of this study was a lack of association between bleeding complications and a more potent platelet inhibition using ticagrelor compared with clopidogrel, in patients with suspected CCS referred for CA. Subgroup analyses restricted to patients undergoing ad hoc PCI supported the findings in the whole population. Moreover, the result was similar in a propensity score-adjusted analysis. Finally, the result was consistent in the as-treated analysis (clopidogrel treated vs. ticagrelor treated) and the “intention-to-treat” analysis (clopidogrel-based period vs. ticagrelor-based period). Our second aim was to assess potential differences in MACE at 30 days with a more potent platelet inhibition. Again, we observed no statistically significant differences.

Although we, in this study on all-comers with suspected CCS and referred for CA, observed no difference in bleeding between the study groups, more than a fifth of the population experienced a bleeding complication. For patients treated with ad hoc PCI, the proportion was more than one in four. Almost all the bleeding events were access site related. The majority of the bleeding complications were classified as TIMI minimal or BARC type 1, but notably, a significant proportion were more severe TIMI minor or BARC type 2.

This is, to our knowledge, the first report on the use of ticagrelor versus clopidogrel, given in a timely fashion to ensure drug effect at the time of CA/PCI, in a real-world setting with CCS patients referred for elective CA, with ad hoc PCI if feasible. In agreement with our finding, the SASSICAIA trial, comparing prasugrel with clopidogrel given immediately before PCI, did not find any significant difference in bleeding complications at 30 days [19]. The ALPHEUS trial, comparing ticagrelor with clopidogrel in elective PCI, also administered immediately before PCI, found no significant difference in bleeding complications at 48 h but more bleeds at 30 days associated with ticagrelor, driven by an increase in BARC 1 and 2 bleeding events [18]. The difference between our finding and those from the ALPHEUS trial may be explained by the fact that most PCI-treated patients in our study were treated with clopidogrel at discharge. We observed a substantially higher bleeding rate in our study (about 20% BARC 1 or 2) compared to the ALPHEUS trial (about 6% BARC 1 or 2). Bleeding complications in our study were entirely driven by access site bleeds, and probably reflect that we included an all-comer population with a higher bleeding risk but also the fact that all patients were pre-loaded with aspirin and a P2Y12 inhibitor and therefore had optimal antiplatelet effect at the time of CA.

In agreement with both the SASSICIA trial and the ALPHEUS trial, we did not observe any significant difference in short-term ischemic events. Of note, we found substantially fewer MIs in our population (3.4%) compared with the SASSICIA trial (16.9%) and the ALPHEUS trial (9%). The difference between these results probably reflects that MIs in our study were diagnosed based on clinical signs and symptoms, and post-PCI troponins were not measured routinely. Nevertheless, neither in our study nor in the SASSICAIA or the ALPHEUS trials (using hs-Tn to detect myocardial damage), were there any significant differences in MI with a more potent P2Y12 inhibitor compared with clopidogrel.

Contrasting our findings, the large observational trial by Li et al. including almost 10,000 patients (but only about 1,000 patients treated with the potent platelet inhibitor ticagrelor) including a propensity score matched analysis, reported a lower MACE rate in ticagrelor-treated patients (which persisted in the propensity matched analysis) and a higher rate of bleeding with ticagrelor (which was no longer significant in the propensity-matched analysis). The observed difference compared with our data may be explained by the longer follow-up time in the study by Li et al. [14, 23, 24] (1 year) and longer-term treatment with ticagrelor. Long-term treatment with ticagrelor has repeatedly shown increased bleeding rates compared with clopidogrel.

Our study adds to current knowledge showing that also when DAPT was administered in a timely fashion to achieve optimal platelet inhibition at the time of PCI, there were no differences in bleeding or ischemic events with potent P2Y12 inhibition compared with clopidogrel in CCS patients referred for CA. The 2010 ESC/EACTS guidelines on revascularization recommended preloading with clopidogrel in patients with CCS, referred for CA. In later revascularization guidelines, and in the CCS guidelines from 2019, preloading was recommended when the anatomy was known, and there was a decision to proceed to PCI (class I) or if the probability of significant coronary artery disease was deemed high (class IIb) [2, 25]. The recommendation was based primarily on two randomized trials showing no benefit with a preloading strategy with clopidogrel, compared with loading in the cath-lab [7, 8]. In a Swedish context, almost 50% of the patients referred for CA because of suspected CCS are treated with ad hoc PCI, which is significantly higher than in the PRAGUE-8 trial comparing preloading to post-CA loading in the cath-lab [7]. It could therefore be argued that, due to a high probability of PCI ad hoc, these patients should be pretreated with DAPT in a timely fashion to ensure adequate platelet inhibition at the time of the procedure. With the similar rate of bleeding complications between a strategy based on the second generation P2Y12 inhibitor clopidogrel and the third generation, more potent, P2Y12 inhibitor ticagrelor, a strategy with preloading at arrival to the outpatient ward would be more convenient. However, we observed a higher rate of bleeding complications compared with previous reports. Even if the bleeding complications were minor, they may well have an impact on patients quality of life and later drug adherence [26, 27]. Moreover, with the observed lack of difference in MACE in our study and especially in previous randomized trials, current knowledge supports the ESC guidelines, not recommending preloading in CCS patients referred for CA. In addition, 109 of 225 (48%) bleeding complications in our study occurred in patients not treated with ad hoc PCI, further supporting a general no preloading approach.

Importantly, there are ways to achieve fast and potent platelet inhibition also with loading dose given after the CA; either using the iv P2Y12 inhibitor cangrelor [17] or using chewed oral ticagrelor. In a study by Venetsanos [28], on patients with CCS referred for CA, a chewed loading dose of ticagrelor (180 mg) resulted in significantly faster platelet inhibition compared with conventional whole tablet administration. Already after 20 min, all patients randomized to chewed ticagrelor achieved adequate platelet inhibition (defined as <208 P2Y12 Reaction Units) as compared with 68% in the group randomized to common practice, swallowing the tablets whole. Therefore, an alternative strategy could be a loading dose of chewed ticagrelor, after the CA when decision to proceed to PCI, which would secure adequate platelet inhibition within less than 20 min.

### Limitations

The strength of this analysis is that it includes an all-comers population with CCS referred for CA. However, the analysis has several limitations that need to be mentioned. First, the observational design comparing two treatments (in the as-treated analysis) or two time periods (in the “intention-to-treat analysis”) may have introduced unknown confounders. The study groups appeared relatively well-balanced regarding baseline characteristics, and in addition, we performed propensity score-adjusted analyses. However, unmeasured confounders cannot be adjusted for. Second, the relatively small study population increase the risk of a type-II error. However, bleeding complications were frequent, occurring in between 20 and 25% of the patients. Third, the study was not powered to detect differences in MACE, an infrequent endpoint in this population. Finally, we did not routinely measure troponins to detect potential differences in in peri-procedural myocardial damage.

## Conclusion

In patients with CCS referred for CA and PCI, if feasible, more than one in five experienced a bleeding complication. A more potent platelet-inhibition strategy, with loading-dose based on ticagrelor, was not associated with an increase in bleeding complications or decrease in ischemic events.

Bleeding complications were common also in patients undergoing CA only. Supporting our main analysis, the sensitivity analysis based on the recommended first-line treatment (“intention-to-treat”) showed no differences in bleeding complications.

## Statement of Ethics

The study was conducted in accordance with the Declaration of Helsinki. In accordance with the ethical regulations for National Swedish quality registries, all patients were informed about their participation in the registry and the right to deny registration, thereby waiving the written informed consent. For the current study, approval was obtained from the Ethical Review Board in Linkoping (approval number: Dnr 2016/325-31).

## Conflict of Interest Statement

Erik Träff, Dimitrios Venetsanos, Karin Alpkvist, Sofia Sederholm Lawesson, Mikolaj Skibniewski, and Sammy Zwackman report no conflict of interests. Joakim Alfredsson reports lecture fees from Boehringer Ingelheim, AstraZeneca, MSD, and Bayer. Joakim Alfredsson has served on Advisory Board for AstraZeneca and Novartis.

## Funding Sources

This work was supported by Linköping University Hospital Research Fund, Linköping, Sweden.

## Author Contributions

Joakim Alfredsson, Erik Träff, Dimitrios Venetsanos, and Karin Alpkvist designed the study, collected data, made the analyses, interpreted data, and wrote the first draft. Sofia Sederholm Lawesson, Mikolaj Skibniewski, and Sammy Zwackman interpreted data and critically revised the draft.

All the authors approved the final version of the manuscript, and agreed to be accountable for all aspects of the work in ensuring that questions related to the accuracy or integrity of any part of the work are appropriately investigated and resolved.

## Data Availability

SWEDEHEART does not allow individual data sharing to third party. Access to aggregated data might be granted following review by the SWEDEHEART Steering Committee. Such requests can be submitted to the SWEDEHEART Steering Committee for consideration. Further inquiries can be directed to the corresponding author.

## Supplementary Material

Supplementary data

## Figures and Tables

**Table 1:** Baseline characteristics

	Clopidogrel (*n* = 577)	Ticagrelor (*n* = 426)	*p* value
Demographics			
Age, years (mean ± SD)	66.5±9.7	66.6±9.4	0.87
Female sex	186 (32.2)	132 (31.0)	0.17
BMI, kg/m^2^ (mean ± SD)	27.8±4.6	27.6±4.5	0.54
Medical history			
Hypertension	401 (69.5)	296 (69.5)	1.00
Diabetes mellitus	162 (28.1)	108 (25.4)	0.34
Previous history of MI	142 (24.6)	94 (22.1)	0.35
Previous history of PCI	137 (23.7)	88 (20.7)	0.25
Previous history of CABG	65 (11.3)	36 (8.5)	0.14
Previous bleeding	91 (15.8)	77 (18.1)	0.33
Previous GI bleeding	38 (6.6)	25 (5.9)	0.64
Smoking			
Current smoker	57 (9.9)	43 (10.1)	0.22
Former smoker	318 (55.1)	212 (49.8)	0.22
CCS angina score			
Class I	28 (5.1)	35 (8.4)	0.12
Class II	328 (59.9)	257 (61.5)	
Clas III	190 (34.7)	125 (29.9)	
Class IV	2 (0.4)	1 (0.2)	
Laboratory findings			
Anemia*	81 (14.0)	31 (7.3)	0.001
Hb on arrival, g/L (mean ± SD)	137.6±12.3	141.5±12.1	<0.001
Platelet count, x10^9^/L (mean ± SD)	244.4±74.4	236.9±66.6	0.097
Creatinine, umol/L (mean ± SD)	91.6±45.9	80.7±21.7	<0.001
eGFR-CG**, mL/min (mean ± SD)	85.1±36.8	91.1±32.0	0.009
CKD** (eGFR <45 mL/min), *n* (%)	54 (9.8)	14 (3.5)	<0.001
CKD** (eGFR <60 mL/min), *n* (%)	108 (19.6)	42 (10.4)	<0.001
Medication prior to angiography			
Aspirin	577 (100.0)	426 (100.0)	N/A
Clopidogrel	577 (100.0)	0 (0.0)	N/A
Ticagrelor	0 (0.0)	426 (100.0)	N/A
Warfarin	42 (7.3)	27 (6.3)	0.56
NOAC	1 (0.2)	4 (0.9)	0.17
Statin	473 (82.0)	311 (73.0)	0.001

Results are presented as numbers (percentages). SD, standard deviation; BMI, body mass index; MI, myocardial infarction; PCI, percutaneous coronary intervention; CABG, coronary artery bypass grafting; GI, gastrointestinal; CCS, Canadian cardiovascular Society; Hb, hemoglobin; eGFR, estimated glomerular filtration rate (calculated using the Cockroft-Gault equation); CKD, chronic kidney disease; NOAC, non-vitamin K antagonist oral anticoagulant.

* Anemia defined as <120 g/L for women and <130 g/L for men.

** Based on 550 of 577 and 403 of 426 patients respectively, with complete information to calculate eGFR-CG.

**Table 2:** Interventions and medication at discharge

	Clopidogrel (*n* = 577)	Ticagrelor (*n* = 426)	*P* value
Interventions			
Radial access	409 (69.8)	331 (77.7)	0.02
Severity of coronary disease			
No sign. coronary disease*	155 (26.9)	94 (22.1)	0.15
1 vessel disease	169 (29.3)	127 (29.8)	
2 vessel disease	101 (17.5)	102 (23.9)	
3 vessel disease	112 (19.4)	78 (18.3)	
Left main stenosis	40 (6.9)	25 (5.9)	
PCI ad hoc	**244 (42.2)**	**200 (46.9)**	0.14
PCI with stent	194 (33.6)	159 (37.3)	0.05
PCI with DEB	51 (8.8)	51 (12.0)	0.04
PCI with DES	165 (28.6)	155 (36.4)	<0.01
PCI including rotablator	3 (0.5)	4 (0.9)	0.05
Diagnostic procedure (FFR, iFR)	**70 (12.1)**	**64 (15.0)**	0.11
CA only	**263 (45.6)**	**162 (38.0)**	0.02
Planned interventions			
Elective PCI	20 (3.5)	24 (5.6)	0.01
Elective CABG	80 (13.9)	44 (10.3)	0.01
Revascularisation total	344 (59.6)	268 (62.9)	0.29
Number of stents			0.21
1 stent	139 (24.1)	100 (23.5)	
2 stents	43 (7.5)	46 (10.8)	
3 or more stents	12 (2.1)	13 (3.1)	
Medication at discharge			
Aspirin	510 (88.4)	377 (88.5)	0.96
Clopidogrel	256 (44.4)	144 (33.8)	<0.01
Ticagrelor	3 (0.5)	46 (10.8)	<0.01
Warfarin	43 (7.5)	40 (9.4)	0.27
NOAC	1 (0.2)	3 (0.7)	0.32
DAPT	247 (42.8)	188 (44.1)	0.68
DAT	0 (0)	1 (0.2)	0.43
TAT	24 (4.2)	16 (3.8)	0.75

Results are presented as numbers and (percentages). PCI, percutaneous coronary intervention; DEB, drug eluting balloon; DES, drug eluting stent; FFR, fractional flow reserve; iFR, instantaneous wave-free ratio; CABG, coronary artery bypass grafting; NOAC, non-vitamin K antagonist oral anticoagulant; DAPT, dual antiplatelet therapy; DAT, dual antithrombotic therapy; TAT, triple antithrombotic therapy.

* Including 4 patients with inconclusive findings (2 in each group).

**Table 3:** Outcomes 30 days

	Clopidogrel	Ticagrelor	*p* value
	(*n* = 577)	(*n* = 426)	
Any Bleeding	132 (22.9)	93 (21.8)	0.70
TIMI minimal	79 (13.7)	52 (12.2)	0.78*
TIMI minor	53 (9.2)	41 (9.6)	
TIMI major	0 (0.0)	0 (0.0)	
BARC type 1	79 (13.7)	52 (12.2)	0.91**
BARC type 2	52 (9.0)	40 (9.4)	
BARC type 3a+3b	1 (0.2)	1 (0.2)	
BARC type 4+5	0 (0.0)	0 (0.0)	
Bleeding localization			
Arterial access site	128 (97.0)	91 (97.8)	0.23
Urogenital	0 (0.0)	1 (1.1)	
Nasal	3 (2.3)	0 (0.0)	
Intramuscular	1 (0.8)	0 (0.0)	
Pericardial	0 (0.0)	1 (1.1)	
MACE 30 days	7 (1.2)	10 (2.3)	0.17
MI	6 (1.0)	10 (2.3)	0.28
Periprocedural MI	6 (1.0)	8 (1.9)	0.26
ST	0 (0.0)	1 (0.2)	0.43
Stroke	1 (0.2)	0 (0.0)	1.0
Death	0 (0.0)	0 (0.0)	N/A

Results are presented as numbers and (percentages). TIMI, thrombolysis in myocardial infarction; BARC, Bleeding Academic Research Consortium; MACE, major adverse cardiovascular events (includes death, stent thrombosis, MI and stroke within 30 days); MI, myocardial infarction.

* Statistical test for all TIMI bleedings.

** Statistical test for all BARC bleedings.

**Table 4:** Outcomes in subgroups

**A** Subgroup analysis in patients treated with PCI ad hoc			
	Clopidogrel	Ticagrelor	*P* value
	(*n* = 244)	(*n* = 200)	
Any Bleeding	62 (25.4)	54 (27.0)	0.70
TIMI minimal	32 (13.1)	28 (14.0)	0.93
TIMI minor	30 (12.3)	26 (13.0)	
TIMI major	0 (0.0)	0 (0.0)	
BARC type 1	32 (13.1)	28 (14.0)	0.79
BARC type 2	29 (11.9)	26 (13.0)	
BARC type 3a+3b	1 (0.4)	0 (0.0)	
BARC type 4+5	0 (0.0)	0 (0.0)	
MACE 30d	7 (2.9)	9 (4.5)	0.36
MI	6 (2.5)	9 (4.5)	0.24
Periprocedural MI	6 (2.5)	8 (4.0)	0.36
ST	0 (0.0)	1 (0.5)	0.45
Stroke	1 (0.4)	0 (0.0)	1.0
Death	0 (0.0)	0 (0.0)	−

**B** Subgroup analysis in patients undergoing CA only or CA with diagnostic procedure			
	**Clopidogrel**	**Ticagrelor**	***p* value**
	**(*n* = 333)**	**(*n* = 226)**	

Any Bleeding	70 (21.0)	39 (17.3)	0.27
TIMI minimal	47 (14.1)	24 (10.6)	0.46*
TIMI minor	23 (6.9)	15 (6.6)	
TIMI major	0 (0.0)	0 (0.0)	
BARC type 1	47 (14.1)	24 (10.6)	0.38**
BARC type 2	23 (6.9)	14 (6.2)	
BARC type 3a+3b	0 (0.0)	1 (0.4)	
BARC type 4+5	0 (0.0)	0 (0.0)	
MACE 30d	0 (0.0)	1 (0.4)	0.40
MI	0 (0.0)	1 (0.4)	
Periprocedural MI	0 (0.0)	0 (0.0)	
ST	0 (0.0)	0 (0.0)	
Stroke	0 (0.0)	0 (0.0)	
Death	0 (0.0)	0 (0.0)	

Results are presented as numbers and (percentages). PCI, percutaneous coronary intervention; TIMI, thrombolysis in myocardial infarction; BARC, Bleeding Academic Research Consortium; MACE, major adverse cardiovascular events (includes death, ST, MI, and stroke within 30 days); MI, myocardial infarction.

* Statistical test for all TIMI bleedings.

** Statistical test for all BARC bleedings.
