# Supplementary material for: Real-World Data on Potent P2Y12 Inhibition in Patients with Suspected Chronic Coronary Syndrome, Referred for Coronary Angiography
Source: Cardiology. 2022 Oct 10;147(5-6):486–96. doi: 10.1159/000527459 (PMC9808708; doi:10.1159/000527459)
Supplement: Supplementary file 1 — Supplementary data [file crd-0147-0486-s01.docx]

| SupplementContentBleeding definitionsTable S1. Baseline characteristics Intention to treat analysisTable S2. Interventions and medication at discharge Intention to treat analysisTable S3. Outcomes Intention to treat analysisTable S4. Outcomes in subgroups Intention to treat analysisBleeding definitionsTIMI bleeding criteria **Minimal**   - Any overt bleeding event that does not meet the criteria below. - Any clinically overt sign of hemorrhage (including imaging) associated with a <3 g/dL decrease in hemoglobin concentration or <9% decrease in hematocrit.   **Minor**   - Clinically overt (including imaging), resulting in hemoglobin drop of 3 to <5 g/dL or ≥10% decrease in hematocrit. - No observed blood loss: ≥4 g/dL decrease in the hemoglobin concentration or ≥12% decrease in hematocrit. - Any overt sign of hemorrhage that meets one of the following criteria and does not meet criteria for a major or minor bleeding event, as defined above. - Requiring intervention (medical practitioner-guided medical or surgical treatment to stop or treat bleeding, including temporarily or permanently discontinuing or changing the dose of a medication or study drug). - Leading to, or prolonging, hospitalization. - Prompting evaluation (leading to an unscheduled visit to a healthcare professional and diagnostic testing, either laboratory or imaging).  Major  - Intracranial bleeding (excluding microhemorrhages <10 mm evident only on gradient-echo MRI). - Clinically overt signs of hemorrhage associated with a drop in hemoglobin of ≥5 g/dL or a ≥15% absolute decrease in hematocrit. - Fatal bleeding (bleeding that directly results in death within 7 d).   **BARC bleeding criteria**  **Type 1**  Bleeding that is not actionable and does not cause the patient to seek unscheduled performance of studies, hospitalization, or treatment by a health-care professional; may include episodes leading to self-discontinuation of medical therapy by the patient without consulting a health-care professional.  **Type 2**  Any overt, actionable sign of hemorrhage (e.g., more bleeding than would be expected for a clinical circumstance, including bleeding found by imaging alone) that does not fit the criteria for type 3, 4, or 5 but does meet at least one of the following criteria:   - Requiring nonsurgical, medical intervention by a health-care professional. - Leading to hospitalization or increased level of care. - Prompting evaluation.   **Type 3a**   - Overt bleeding plus hemoglobin drop of 3 to < 5 g/dL (provided hemoglobin drop is related to bleed). - Any transfusion with overt bleeding.  Type 3b  - Overt bleeding plus hemoglobin drop ≥5 g/dL (provided hemoglobin drop is related to bleed). - Cardiac tamponade. - Bleeding requiring surgical intervention for control (excluding dental/nasal/skin/hemorrhoid). - Bleeding requiring intravenous vasoactive agents.   **Type 3c**   - Intracranial hemorrhage (does not include microbleeds or hemorrhagic transformation, does include intraspinal). - Subcategories confirmed by autopsy or imaging or lumbar puncture. - Intraocular bleed compromising vision.   **Type 4**   - CABG-related bleeding. - Perioperative intracranial bleedning within 48h. - Reoperation after closure of sternotomy for the purpose of controlling bleeding. - Transfusion of ≥ 5 U whole blood or packed red blood cells within a 48h period. - Chest tube output more than or equal to 2L within a 24h period.   **Type 5**   - Fatal bleeding.  Type 5aProbable fatal bleeding; no autopsy or imaging confirmation but clinically suspicious.Type 5b  - Definite fatal bleeding; overt bleeding or autopsy or imaging confirmation. | | | |
| --- | --- | --- | --- |
|  | | | |
|  | | | |
| Table S1. Baseline characteristics Intention to treat analysis | | | |
|  | **Clopidogrel-based  strategy**  (n = 502) | **Ticagrelor-based strategy**  (n = 502) | p-value |
| **Demographics** |  |  |  |
| Age, year (mean ± SD) | 65.9 ± 9.3 | 67.2 ± 9,8 | 0.04 |
| Female sex | 164 (32.7) | 154 (30.7) | 0.50 |
| Body weight, kg (mean ± SD) | 83.4 ± 15.6 | 83.1 ± 15.1 | 0.78 |
| BMI kg/m^2^ (mean ± SD) | 27.8 ± 4.5 | 27.7 ± 4 .5 | 0.88 |
| **Medical History** |  |  |  |
| Hypertension | 340 (67.7) | 358 (71.3) | 0.22 |
| Diabetes Mellitus | 130 (25.9) | 140 (27.9) | 0.48 |
| Previous history of MI | 117 (23.3) | 119 (23.7) | 0.88 |
| Previous history of PCI | 111 (22.1) | 114 (22.7) | 0.82 |
| Previous history of CABG | 54 (10.8) | 47 (9.4) | 0.46 |
| Previous bleeding | 73 (14.5) | 95 (18.9) | 0.06 |
| Previous GI bleeding | 32 (6.4) | 31 (6.2) | 0.90 |
| Current smoker | 51 (10.2) | 50 (10.0) | 0.25 |
| Former smoker | 277 (55.2) | 253 (50.4) |  |
| **Laboratory findings** |  |  |  |
| Hb on arrival, g/L (mean ± SD) | 138.5 ± 12.2 | 140.1 ± 12.6 | 0.04 |
| Anemia* | 60 (12.0) | 52 (10.4) | 0.42 |
| Platelet count, x10^9^/L (mean ± SD) | 245.5 ± 77.0 | 237.1 ± 64.7 | 0.06 |
| Creatinine, umol/l (mean ± SD) | 87.2 ± 34.9 | 86.6 ± 40.8 | 0.81 |
| eGFR-CG**, ml/min (mean ± SD) | 87.8 ± 36.5 | 87.6 ± 33.4 | 0.93 |
| Renal failure** (eGFR < 45 umol/l) | 33 (6.9) | 35 (7.4) | 0.76 |
| Renal failure** (eGFR < 60 umol/l) | 74 (15.4) | 76 (16.0) | 0.79 |
| **Medication prior to angiography** |  |  |  |
| Aspirin | 502 (100.0) | 502 (100.0) | - |
| Clopidogrel | 489 (97.4) | 88 (17.5) | <0.001 |
| Ticagrelor | 12 (2.4) | 414 (82.5) | <0.001 |
| Prasugrel | 1 (0.2) | 0 (0.0) | N/A |
| Warfarin | 33 (6.6) | 36 (7.2) | 0.71 |
| NOAK | 0 (0) | 5 (1.0) | 0.31 |
| Statin | 410 (81.7) | 375 (74.7) | 0.02 |
| Results are presented as numbers and percentages unless otherwise indicated. Abbreviations (in order of appearance): SD, standard deviation; BMI, body mass index; MI, myocardial infarction; PCI, percutaneous coronary intervention; CABG, coronary artery by-pass grafting; GI, gastrointestinal; NOAC, non-vitamin K antagonist oral anticoagulant; Hb, Hemoglobin; eGFR, estimated glomerular filtration rate (calculated with the Cockroft-Gault equation); N/A, not applicable. * Anemia defined as <120 g/L for female and < 130 g/L for male. ** Based on 480 of 502 and 474 of 502 patients respectively, with complete information to calculate eGFR-CG. | | | |

| **Table S2. Interventions and medication at discharge Intention to treat analysis** | | | |
| --- | --- | --- | --- |
|  | **Clopidogrel-based  strategy**  (n = 502) | **Ticagrelor-based strategy**  (n = 502) | p-value |
| **Interventions** |  |  |  |
| Radial access | 353 (70.3) | 382 (76.1) | 0.12 |
| Severity of coronary disease |  |  | 0.84 |
| No sign. coronary disease* | 133 (26.5) | 116 (23.1) |  |
| 1 vessel disease | 145 (28.9) | 152 (30.3) |  |
| 2 vessel disease | 96 (19.1) | 107 (21.3) |  |
| 3 vessel disease | 94 (18.7) | 96 (19.1) |  |
| Left main stenosis | 34 (6.8) | 31 (6.2) |  |
| PCI ad hoc | 206 (41.0) | 239 (47.6) | 0.04 |
| PCI with stent | 165 (32.9) | 188 (37.5) | 0.01 |
| PCI with DEB | 41 (8.2) | 62 (12.4) | 0.004 |
| PCI with DES | 136 (27.1) | 184 (36.7) | <0.001 |
| PCI including rotablator | 2 (0.4) | 5 (1.0) | 0.07 |
| Diagnostic procedure (FFR, IFR) | 60 (12.0) | 74 (14.7) | 0.02 |
| Coronary angiography only | 236 (47%) | 189 (37.6) | 0.003 |
| Planned interventions |  |  |  |
| Elective PCI | 19 (3.8) | 25 (5.0) | 0.02 |
| Elective CABG | 72 (14.3) | 52 (10.4) | 0.02 |
| Revascularisation total | 297 (59.2) | 316 (62.9) | 0.22 |
| Number of stents |  |  | 0.11 |
| 1 stent | 123 (24.5) | 116 (23.1) |  |
| 2 stents | 35 (7.0) | 54 (10.8) |  |
| 3 or more stents | 7 (1.4) | 18 (3.6) |  |
| **Medication at discharge,** |  |  |  |
| Aspirin | 456 (90.8) | 432 (86.1) | 0.01 |
| Clopidogrel | 208 (41.6) | 191 (38.0) | 0.25 |
| Ticagrelor | 3 (0.6) | 46(9.2) | <0.001 |
| Prasugrel | 1 (0.2) | 0 (0.0) | N/A |
| Warfarin | 30 (6.0) | 53 (10.6) | 0.01 |
| NOAC | 0 (0) | 4 (0.8) | 0.05 |
| DAPT | 208 (41.4) | 228 (45.4) | 0.23 |
| DAT | 0 (0) | 1 (0.2) | N/A |
| TAT | 17 (3.4) | 23 (4.6) | 0.33 |
| Results are presented as numbers and (percentages). Abbreviations (in order of appearance): PCI, percutaneous coronary intervention; DEB, drug eluting balloon; DES, drug eluting stent; FFR, fractional flow reserve; iFR, instantaneous wave-free ratio; CABG, coronary artery by-pass grafting; NOAC, non-vitamin K antagonist oral anticoagulant; DAPT, dual antiplatelet therapy; DAT, dual antithrombotic therapy; TAT, triple antithrombotic therapy; N/A, not applicable. *Including inconclusive findings=4. | | | |

| **Table S3. Outcomes Intention to treat analysis** | | | |
| --- | --- | --- | --- |
|  | **Clopidogrel based period** | **Ticagrelor based period** |  |
|  | (n = 502) | (n = 502) | p-value |
|  |  |  |  |
| **Any Bleeding** | 108 (21.5) | 117 (23.3) | 0.50 |
| TIMI minimal | 61 (12.2) | 70 (13.9) | 0.61* |
| TIMI minor | 47 (9.4) | 47 (9.4) |  |
| TIMI major | 0 (0.0) | 0 (0.0) |  |
| BARC type 1 | 61 (12.2) | 70 (13.9) | 0.32** |
| BARC type 2 | 47 (9.4) | 45 (9.0) |  |
| BARC type 3a+3b | 0 (0.0) | 2 (0.4) |  |
| BARC type 4+5 | 0 (0.0) | 0 (0.0) |  |
| **Bleeding localisation** |  |  | 0.50 |
| Arterial access site | 105 (97.2) | 114 (97.4) |  |
| Urogenital | 0 (0.0) | 1 (0.9) |  |
| Nasal | 2 (1.9) | 1 (0.9) |  |
| Intramuscular | 1 (1.0) | 0 (0.0) |  |
| Pericardial | 0 (0.0) | 1 (0.9) |  |
| **MACE 30 days** | 6 (1.2) | 11 (2.2) | 0.22 |
| Myocardial infarction | 5 (1.0) | 11 (2.2) | 0.13 |
| Periprocedural MI | 5 (1.0) | 9 (1.8) | 0.28 |
| Stent thrombosis | 0 (0.0) | 1 (0.2) | N/A |
| Stroke | 1 (0.2) | 0 (0.0) | N/A |
| Death | 0 (0.0) | 0 (0.0) | N/A |
| Results are presented as numbers and (percentages). Abbreviations (in order of appearance): MACE, major adverse cardiovascular events (includes death, stent thrombosis, myocardial infarction and stroke at 30 days); MI, myocardial infarction; TIMI, Thrombolysis in Myocardial Infarction; BARC, Bleeding Academic Research Consortium; N/A, not applicable.  * Statistical test for all TIMI bleedings. ** Statistical test for all BARC bleedings. | | | |

| **Table S4. Outcomes in subgroups Intention to treat analysis** | | | |
| --- | --- | --- | --- |
| **4a. Subgroup analysis in patients treated with PCI ad hoc** | | | |
|  | **Clopidogrel based period** | **Ticagrelor based period** |  |
|  | (n = 206) | (n = 239) | p-value |
|  |  |  |  |
| **Any Bleeding** | 49 (23.8) | 67 (28.0) | 0.31 |
| TIMI minimal | 23 (11.2) | 37 (15.5) | 0.38 |
| TIMI minor | 26 (12.6) | 30 (12.6) |  |
| TIMI major | 0 (0.0) | 0 (0.0) |  |
| BARC type 1 | 23 (11.2) | 37 (15.5) | 0.43 |
| BARC type 2 | 26 (12.6) | 29 (12.1) |  |
| BARC type 3a+3b | 0 (0.0) | 1 (0.4) |  |
| BARC type 4+5 | 0 (0.0) | 0 (0.0) |  |
| **MACE 30d** | 6 (2.9) | 10 (4.2) | 0.47 |
| Myocardial infarction | 5 (2.4) | 10 (4.2) | 0.31 |
| Periprocedural MI | 5 (2.4) | 9 (3.8) | 0.42 |
| Stent thrombosis | 0 (0.0) | 1 (0.4) | 1.0 |
| Stroke | 1 (0.5) | 0 (0.0) | 0.46 |
| Death | 0 (0.0) | 0 (0.0) | - |
| **4b. Subgroup analysis in patients undergoing CA only or CA with diagnostic procedure.** | | | |
|  | **Clopidogrel based group** | **Ticagrelor based group** |  |
|  | (n =296) | (n = 263) | p-value |
|  |  |  |  |
| **Any Bleeding** | 59 (19.9) | 50 (19.0) | 0.78 |
| TIMI minimal | 38 (12.8) | 33 (12.5) | 0.95 |
| TIMI minor | 21 (7.1) | 17 (6.5) |  |
| TIMI major | 0 (0.0) | 0 (0.0) |  |
| BARC type 1 | 38 (12.8) | 33 (12.5) | 0.71 |
| BARC type 2 | 21 (7.5) | 16 (6.1) |  |
| BARC type 3a+3b | 0 (0.0) | 1 (0.4) |  |
| BARC type 4+5 | 0 (0.0) | 0 (0.0) |  |
| **MACE 30d** | 0 (0) | 1 (0.4) | 0.47 |
| Myocardial infarction | 0 (0) | 1 (0.4) | 0.47 |
| Periprocedural MI | 0 (0) | 0 (0) | NA |
| Stent thrombosis | 0 (0) | 0 (0) | NA |
| Stroke | 0 (0) | 0 (0) | NA |
| Death | 0 (0) | 0 (0) | NA |
| Results are presented as numbers and (percentages). Abbreviations (in order of appearance): PCI, percutaneous coronary intervention; TIMI, Thrombolysis in Myocardial Infarction; BARC, Bleeding Academic Research Consortium; MACE, major adverse cardiovascular event (includes death, stent thrombosis, myocardial infarction and stroke at 30 days); MI, myocardial infarction; CA, coronary angiography; N/A, not applicable. * Statistical test for all TIMI bleedings. ** Statistical test for all BARC bleedings. | | | |

|  |
| --- |
